# Integrative Bioinformatics and Machine Learning Approaches for Biomarker and Pathway Discovery in Malignant Pleural Mesothelioma

**DOI:** 10.1002/cnr2.70731

**Published:** 2026-10-26

**Authors:** Md. Lotifur Rohman, Suman Biswas

**Affiliations:** ^1^ Department of Statistics and Data Science Islamic University Kushtia Bangladesh

**Keywords:** differentially expressed genes, machine learning, malignant pleural mesothelioma, survival analysis, weighted gene co‐expression network analysis

## Abstract

**Background:**

Malignant pleural mesothelioma (MPM) is a highly aggressive thoracic cancer with limited treatment options and poor prognosis.

**Aims:**

This study employed an integrative transcriptomic and computational approach to uncover potential biomarkers for MPM.

**Methods:**

Gene expression data from the GSE51024 dataset, comprising 41 normal and 55 MPM tumor samples, were preprocessed using the robust multiarray average (RMA) method. Differentially expressed genes (DEGs) were identified using the Limma package and Wilcoxon rank tests. Weighted gene co‐expression network analysis (WGCNA) revealed significant gene modules using topological overlap and preservation statistics, followed by functional annotation through Gene Ontology (GO) and KEGG pathway analyses.

**Results:**

Hub genes within conserved modules (MM > 0.8) were identified, including ZNF638, RAB18, SOS1, TTC17, RANBP2, FNDC3B, QSER1, CLDN15, PSTPIP1, SEPTIN6, ADAP2, and PVRIG. Survival analysis validated their prognostic relevance using public databases. Furthermore, high classification accuracy was achieved using various machine learning algorithms (SVM, PLDA, XGB, K‐NN, and logistic regression), confirming the discriminative potential of the hub genes. The results highlighted several physiologically significant hub genes and pathways associated with cell cycle regulation, immune modulation, and cancer progression.

**Conclusion:**

Overall, this exploratory study demonstrated that potential biomarkers in MPM can be effectively identified using an integrative systems biology and machine learning framework. The identified hub genes may support future diagnostic, prognostic, and personalized treatment strategies for patients with MPM.

**Abbreviations**
DEG: differentially expressed genes
MPM: malignant pleural mesothelioma
NSCLC: nonsmall cell lung cancer
WGCNA: weighted gene co‐expression network analysis

## 1 Introduction

The rare but aggressive cancer known as MPM is primarily associated with asbestos exposure and develops from the mesothelial cells lining the pleura. Due to the extended latency period of MPM and continuous exposure in some areas, the incidence of the disease persists despite legislative efforts to restrict its usage [1]. Due in large part to difficulties in early detection and a lack of effective treatment options, MPM patients typically have a dismal prognosis, with median survival durations ranging from 9 to 17 months after diagnosis [2].

To identify reliable biomarkers for MPM, several molecular candidates have been investigated. Soluble mesothelin‐related peptides (SMRP), fibulin‐3, and osteopontin are examples of protein‐based biomarkers whose diagnostic and prognostic potential has been studied. Regarding early identification or differentiating MPM from other pleural illnesses, these markers frequently have low sensitivity and specificity [2, 3]. For example, although SMRP has shown promise, its diagnostic specificity is diminished because its levels can also be increased in other illnesses.

Differentially expressed genes (DEGs) associated with MPM can now be identified through transcriptome analyses, enabled by advances in high‐throughput technology. Research has identified genes associated with immune responses, apoptosis, and cell cycle regulation [4]. Nonetheless, this research frequently relies on conventional statistical techniques that may miss the intricate relationships within gene networks, leading to results that conflict with previous studies [5].

Integrative bioinformatics techniques have been employed to overcome the limitations of single‐gene analyses. One such technique is weighted gene co‐expression network analysis (WGCNA), which builds gene networks using expression data to find modules of co‐expressed genes that might be functionally connected [6]. WGCNA has been effectively used to identify important regulatory genes and pathways in a variety of malignancies [7].

In addition to WGCNA, machine learning methods provide strong tools for gene expression profile‐based classification and prediction. Predictive models for cancer diagnosis and prognosis have been developed using algorithms like support vector machines (SVM), linear discriminant analysis (LDA), negative binomial discriminant analysis, Naïve Bayes, and logistic regression [8, 9]. These models can handle high‐dimensional data, revealing patterns that traditional analysis might miss.

Despite these developments, few studies have combined machine learning with WGCNA to find and validate reliable biomarkers for MPM. Few studies have found gene signatures that are both biologically and clinically relevant by combining network‐based analyses with predictive modeling. Therefore, the goal of this study is to identify new biomarkers and pathways associated with MPM using advances in machine learning, which could lead to better therapeutic approaches and classification approaches.

## 2 Materials and Methods

### 2.1 Microarray Gene Expression Datasets

The gene expression profiles of MPM tumor and paired normal lung tissue samples from Gene Expression Omnibus (GEO) under accession number GSE51024 were retrieved in this study [10]. The Affymetrix Human Genome U133 Plus 2.0 Array was used to measure the expression levels of 54 675 probe sets. This dataset, deposited by Suraokar and Wistuba [11], contained 96 samples, including 55 tumor samples and 41 paired normal lung samples (see Table S1). The study examined the DEGs and distinct expression patterns between mesothelioma tumors and normal nontumor tissues.

### 2.2 Data Preprocessing and Differential Gene Expression Analysis

There were 54 675 probe sets in the transcriptomics dataset of mesothelioma, GSE51024. The gene expression levels across samples were normalized using the robust multiarray averaging (RMA) approach, as the dataset was nonnormal.

To identify DEGs from the data, the Limma package [12] and the Wilcoxon rank test [13] were carried out in the R programming language. The adjusted *p*‐value with Benjamini–Hochberg FDR correction techniques (FDR < 0.01) and $|\log_{2}\left(\mathrm{FC}\right)|\geq 1$ statistical threshold parameters for DEGs identification were considered, respectively. Thereafter, the set of expressed genes was finalized by taking the intersection of the genes selected by these two methods (Figure 1).

**FIGURE 1:**
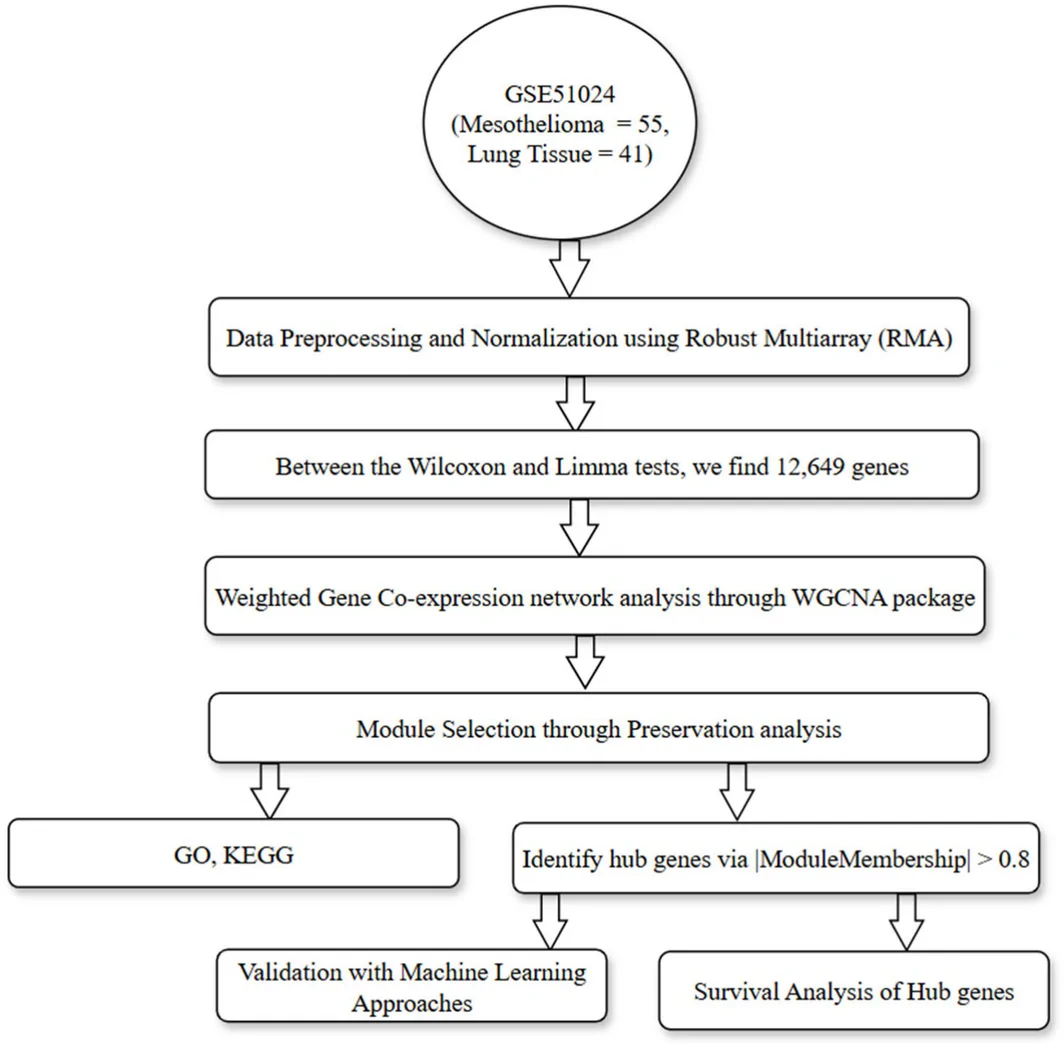
Flowchart of this study.

### 2.3 Weighted Gene Co‐Expression Network Construction

The cluster dendrogram of the samples was constructed using the hclust function in R to identify outliers. The WGCNA package in R [14] was employed to construct the weighted gene co‐expression network. For finding numerous soft‐thresholding powers β over $R^{2}$, the *pickSoftThreshold* function was used, and the value of β was picked for which the value of $R^{2}$ was maximum. The adjacency matrix and topological overlap matrix (TOM) were then constructed from the transformed gene expression matrix using this soft‐threshold power. TOM dissimilarity (dissTOM) was computed to construct a network heatmap. To detect the module, a gene dendrogram was constructed from a dissTOM matrix and the hclust function, and the results were visualized using different colors (Figure 1).

### 2.4 Preservation Analysis for the Key Module Selection

The important modules were identified using module preservation analysis. The unit the *modulePreservation* function [15], was employed to assess the resilience and reproducibility of each module across different data sets. Modules are deemed preserved if preservation statistics *Z*‐summary > 10 [16]. There is a positive association between *Z*‐summary statistics and module preservation; however, the median rank of the preservation statistics is negatively correlated with module preservation [17].

### 2.5 Gene Ontology and Pathway Enrichment Analysis

The high gene interconnection among co‐expression modules may provide important clues regarding the related biological functions of the same module. Gene Ontology (GO) and pathway analysis were used to examine the functional enrichment analysis of the genes in each of the chosen major modules [18]. The GO and KEGG (Kyoto Encyclopedia of Genes and Genomes) pathway enrichment analysis was performed using the Database for Annotation, Visualization, and Integrated Discovery (DAVID) tools [19], and the results were visualized through the *clusterProfiler* package in R [14]. Significant KEGG pathways and GO keywords were chosen using a statistical threshold criterion with an adjusted *p*‐value < 0.05.

### 2.6 Identification of Hub Genes

Genes with the highest level of connectivity within a module were identified as hub genes, representing the core features and connectivity of that module. The absolute value of Pearson's correlation (∣cor.geneModuleMembership∣>0.8) [16] was used to identify the hub genes (Figure 1).

### 2.7 Survival Analysis of Hub Genes

To evaluate their possible impact on patient survival for the diseases or conditions under study, survival curves for important hub genes were then created using a web‐based platform, GEPIA2, for large‐scale gene expression [20].

### 2.8 Performance Evaluation of the Hub Genes With Classification Algorithms

To evaluate the within‐cohort discriminatory potential of the identified hub‐gene panel, five supervised machine learning classifiers were applied: SVM [21] with radial basis kernel function, LDA [22], logistic regression (LR) [23], eXtreme gradient boosting (XGB) [24], and *K*‐nearest neighbor (KNN) [25]. Repeated stratified fivefold cross‐validation [26] with 25 repeats was used to evaluate the model's performance. Within the resampling architecture, hyperparameters were optimized for every classifier. Accuracy, area under the receiver operating characteristic curve (AUC), sensitivity, and specificity—all expressed as mean ± standard deviation across resamples—were used to summarize classification performance. Additionally, 95% confidence intervals for the AUC were obtained using pooled out‐of‐fold ROC predictions [27].

## 3 Analytical Findings

### 3.1 Identification of the DEGs in MPM

There were 54 675 probe sets in the discovery dataset (GSE51024), which included 41 normal lung tissue control samples and 55 mesothelioma tumor samples after normalization (Table 1). A total of 13 305 DEGs were found from this dataset using the Limma Package (FDR < 0.01), and 14 491 DEGs were found using the Wilcoxon test (adj. *p*‐value ≤0.01). Later, 12 649 genes shared by both the Limma Package and the Wilcoxon rank test were identified and considered for subsequent WGCNA. Figure 2 presents the information regarding the datasets used in this study.

**TABLE 1:** Overview of the mesothelioma dataset used in this study.

Methods	Identified DEGs	Selection criteria	Tumor samples	Normal samples
Wilcoxon	14 491	Adj. *p*‐value	55	41
Limma package	13 305	FDR value		

**FIGURE 2:**
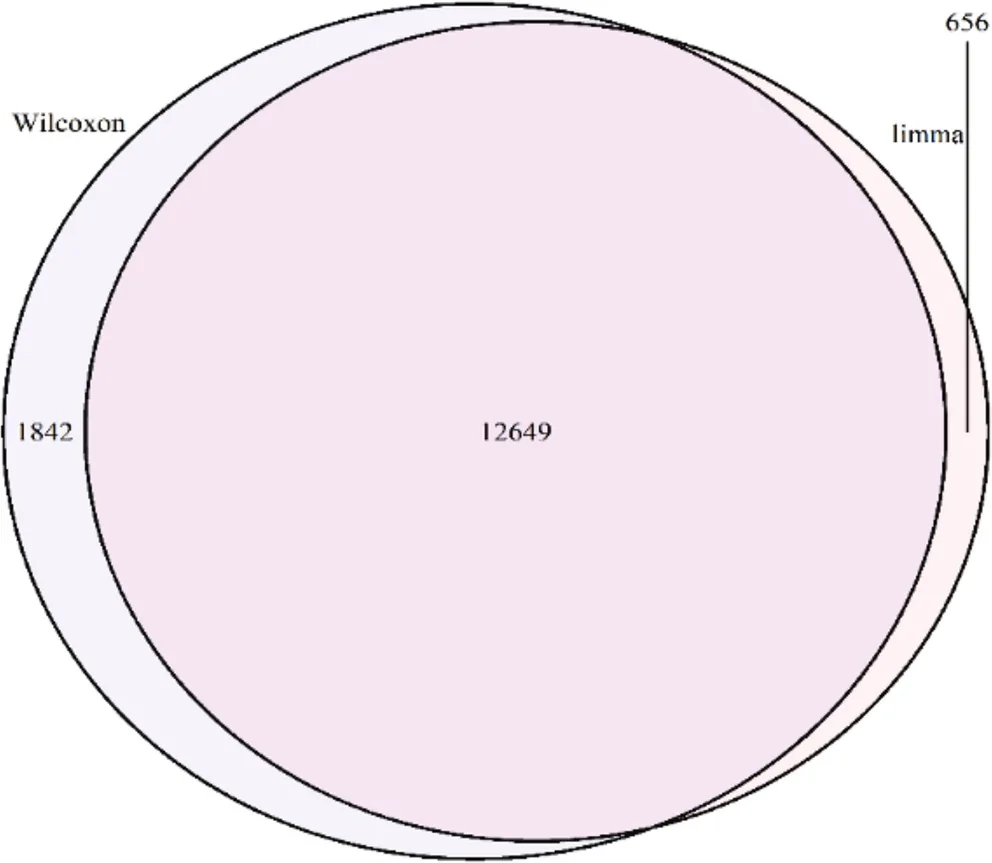
Overlap between differentially expressed genes (DEGs) identified by the Limma and Wilcoxon analyses.

### 3.2 Weighted Gene Coexpression Networks and Module Preservation Analysis

The gene coexpression network analyses were performed with the identified 12 649 DEGs of 96 Mesothelioma samples in the GSE51024 dataset. The cluster dendrogram of these samples, as visualized in Figure 3A, revealed that no outlying samples were present in this dataset. As the scale‐free topology criterion, we chose the optimal soft‐thresholding power, β=9, with $R^{2}=0.90$ (Figure 3B).

**FIGURE 3:**
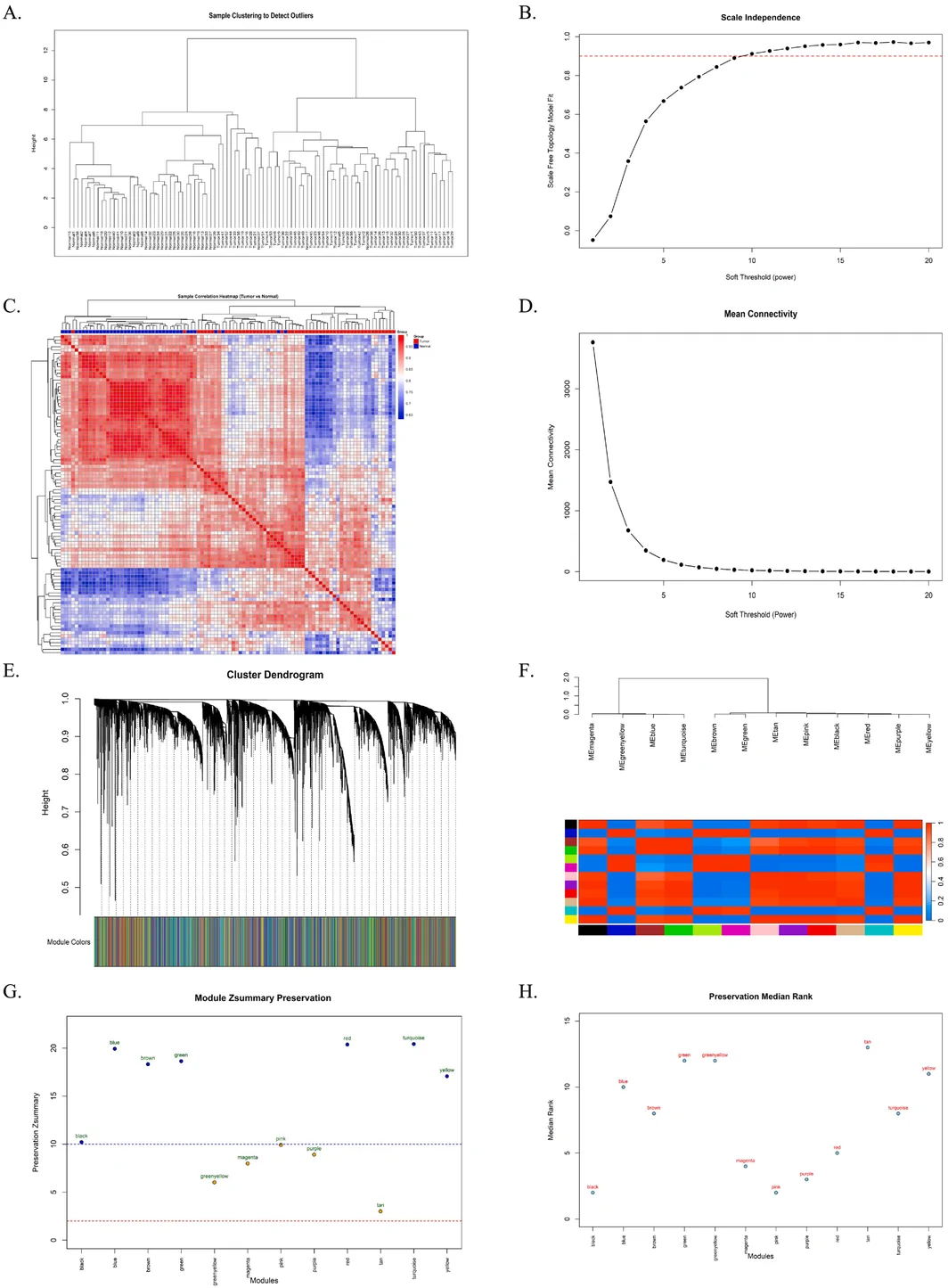
WGCNA co‐expression module construction and hub module selection. (A) The cluster dendrogram of samples afflicted by mesothelioma. (B) Scale‐free fit index for different soft—thresholding powers. (C) Gene Clustering Dendrogram with module. (D) Examine the mean connectivity. (E) Dendrogram of all differentially expressed genes clustered based on a dissimilarity measure (1‐TOM). (F) Heatmap plot of all hub genes. (G) The red and blue dashed lines on the Z summary statistics map across each module represent the moderate and strong preservation criteria, respectively, Z=2andZ=10. (H) The module median rank: a number near zero denotes a high level of module preservation.

The entire WGCNA method for locating co‐expressed gene modules and choosing hub modules is shown in Figure 3. A sample cluster dendrogram, a vital first step for identifying and excluding outlier samples that can distort the results, is shown in Figure 3A. The hierarchical clustering of samples according to their gene expression profiles is shown in this dendrogram. Determining the proper soft‐thresholding power (*β*), a crucial parameter in WGCNA for building a scale‐free network, requires the use of Figure 3B,C.

A soft‐thresholding power that best approximates a scale‐free topology is chosen based on Figure 3C, which appears to be a sample correlation heatmap, and Figure 3B, which plots the scale‐free fit index against various powers. An *R* ^2^ score near 1.0 usually indicates a good fit. A soft‐thresholding power that promotes scale‐free features and preserves high network connectivity is chosen using Figure 3D, which shows the Mean connection. The hierarchical clustering of genes based on the topological overlap measure (TOM) is shown in Figure 3E, “Cluster Dendrogram,” in which genes with comparable expression patterns are grouped together. The detected co‐expression modules are shown by the colored bars beneath the dendrogram; each color denotes a different module. Module eigengenes (ME) can summarize a module's overall expression profile. They are the initial principal components of each module's gene expression.

A module preservation study was carried out using the dataset (GSE51024, which contains 41 normal samples) as the test dataset to confirm the stability and applicability of the found modules. Figure 3G,H summarize the findings. Figure 3G, the module *Z*‐summary Preservation plot, provides a statistical measure of module preservation. The red dashed line at Z=2 and the blue dashed line at Z=10 represent thresholds for moderate and strong preservation, respectively. Modules with a *Z*‐summary statistic above 10 are considered strongly preserved, while those between 2 and 10 are moderately preserved. Based on the *Z*‐summary preservation criterion, the black, pink, and red modules showed strong preservation. In contrast, the magenta and purple modules did not reach the *Z*‐summary > 10 threshold for strong preservation but showed favorable relative preservation according to medianRank.

The Preservation Median Rank plot in Figure 3H assesses module preservation using their rankings. Greater preservation is indicated by a lower median rank, implying that the module is well preserved in both datasets. The modules that are most well‐preserved in this figure are the black, magenta, pink, purple, and red ones, which have the lowest median ranks. Therefore, based on the combined evidence from the preservation metrics (Figure 3G,H), the black, pink, purple, magenta, and red modules were identified as moderately stable and well‐preserved.

### 3.3 Pathway and GO Analysis

The significantly DEGs were subjected to Pathway and Gene Ontology (GO) analyses to reveal shared biological pathways and functional categories. For these, the *ClusterProfiler* R package [14] was used in the study. For the common genes, the top 10 KEGG pathways (Figure 4A) and important enrichment terms (Figure 4B) were determined. Table S2 provides further insights into the biological functions of each module by presenting KEGG pathway analyses for each of the five separate modules in addition to the main enrichment results. These findings highlighted pathways that are specifically enriched within each module, complementing the common DEG enrichment. Therefore, the additional information was used to support the biological significance and robustness of the findings.

**FIGURE 4:**
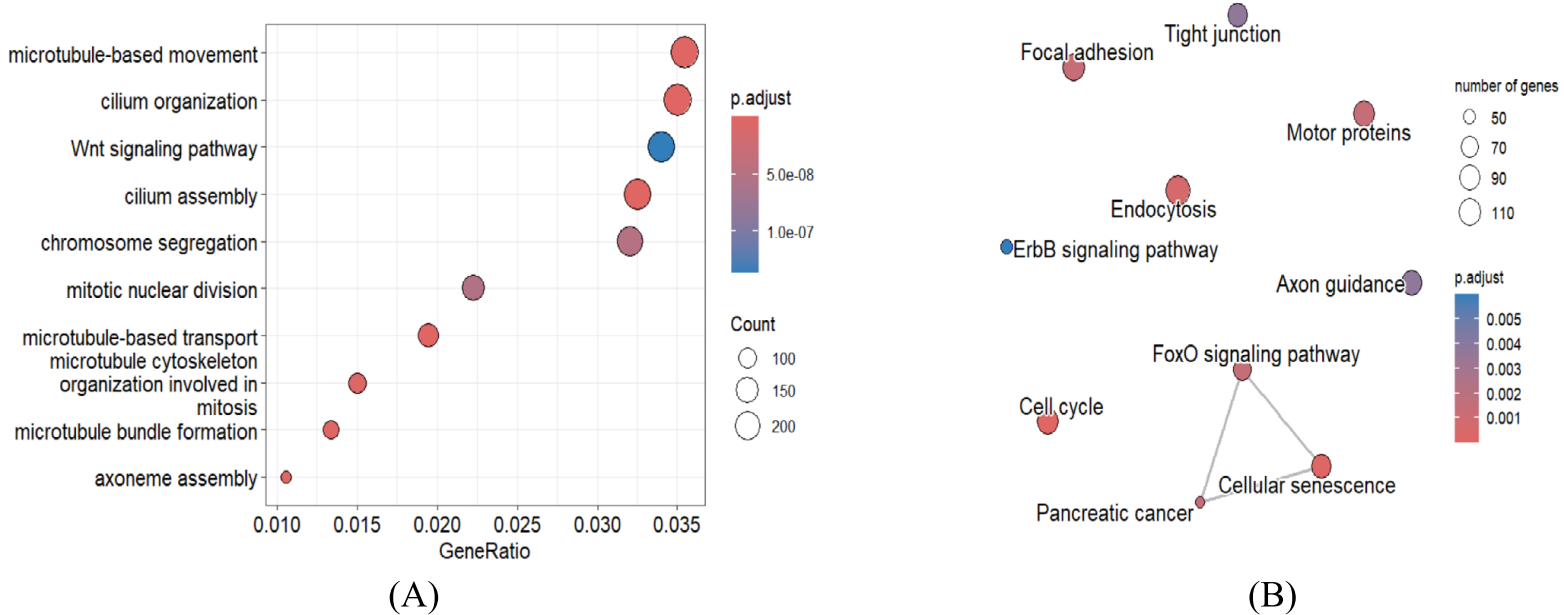
Functional enrichment analysis of shared genes: (A) Gene Ontology and (B) KEGG pathways.

### 3.4 Identification of Hub Genes

A module membership (MM) analysis was conducted on the selected co‐expression modules from the WGCNA network to identify biologically significant hub genes. Hub genes were systematically extracted and annotated to produce a more curated collection of candidates for additional survival analysis and evaluation of hub gene performance. In this study, hub genes were defined as those with an absolute MM (|MM|) > 0.8, indicating a strong association with the corresponding module eigengenes. Five modules of interest—black, magenta, purple, red, and pink—were examined, and hub genes were extracted in accordance with this criterion. Subsequently, probe IDs were mapped to their respective gene symbols using the *hgu133plus2.db* database (Table S3).

After assessing, five hub genes—ZNF638, RAB18, SOS1, TTC17, and RANBP2—were found in the black module, while CLDN15 was present in the purple module, but FNDC3B and QSER1 were contributed by the magenta module. Similarly, four hub genes—PSTPIP1, SEPTIN6, ADAP2, and PVRIG—were identified by the red module. Importantly, no hub genes were identified in the pink module (Figure 5).

**FIGURE 5:**
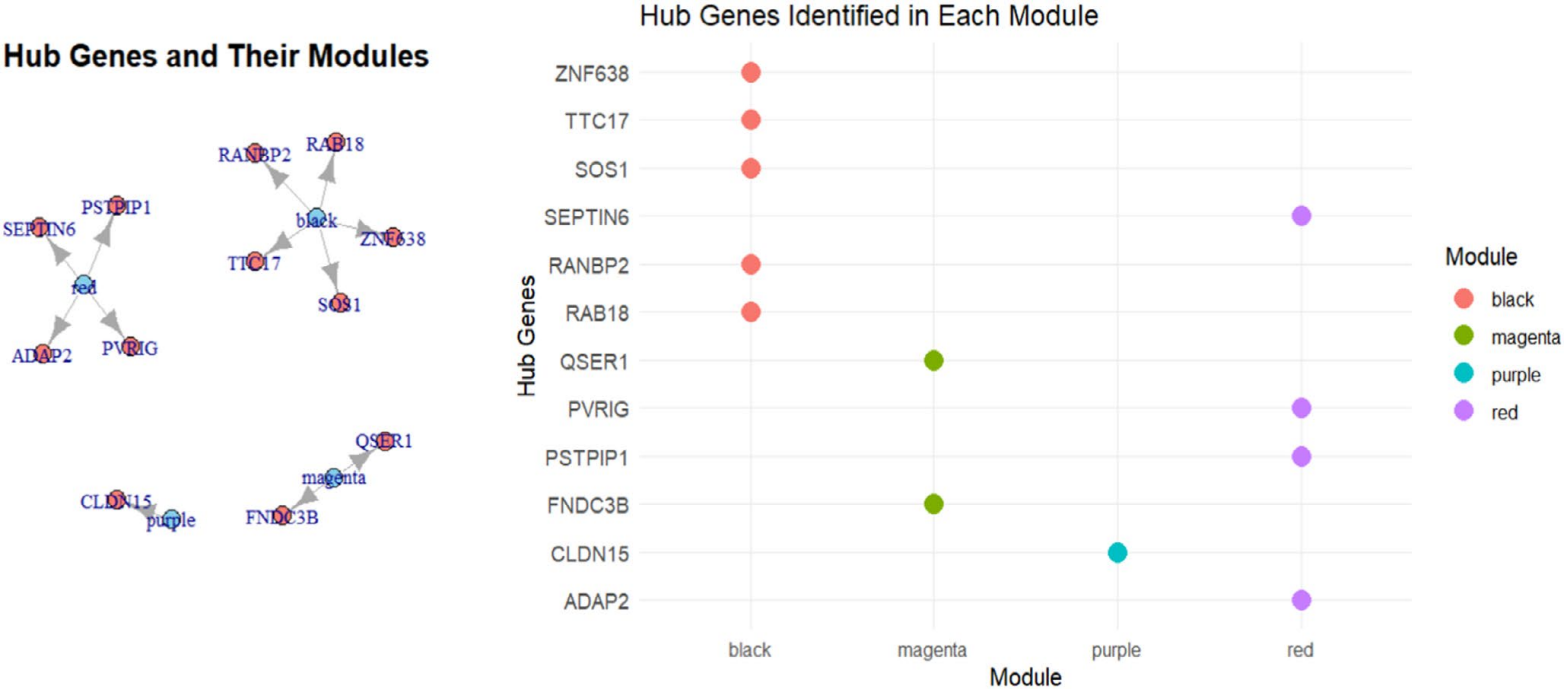
Identified hub genes in each module.

### 3.5 Survival Analysis of Hub Genes

The prognostic relevance of the identified hub genes was evaluated using GEPIA2, a web‐based platform for TCGA‐ and GTEx‐based gene‐expression and survival analysis [20]. To ensure disease‐matched survival assessment, the TCGA mesothelioma cohort (MESO) was selected. The expression profiles of the 12 hub genes—ZNF638, RAB18, SOS1, TTC17, RANBP2, FNDC3B, QSER1, CLDN15, PSTPIP1, SEPTIN6, ADAP2, and PVRIG—were analyzed separately for overall survival. Patients were stratified into high‐ and low‐expression groups using the median expression cutoff, and Kaplan–Meier survival curves were generated. Survival differences were assessed using the log‐rank test, and hazard ratios with 95% confidence intervals were obtained from GEPIA2. The analysis showed that 11 of the 12 identified hub genes were associated with overall survival in the MESO cohort (Figure 6).

**FIGURE 6:**
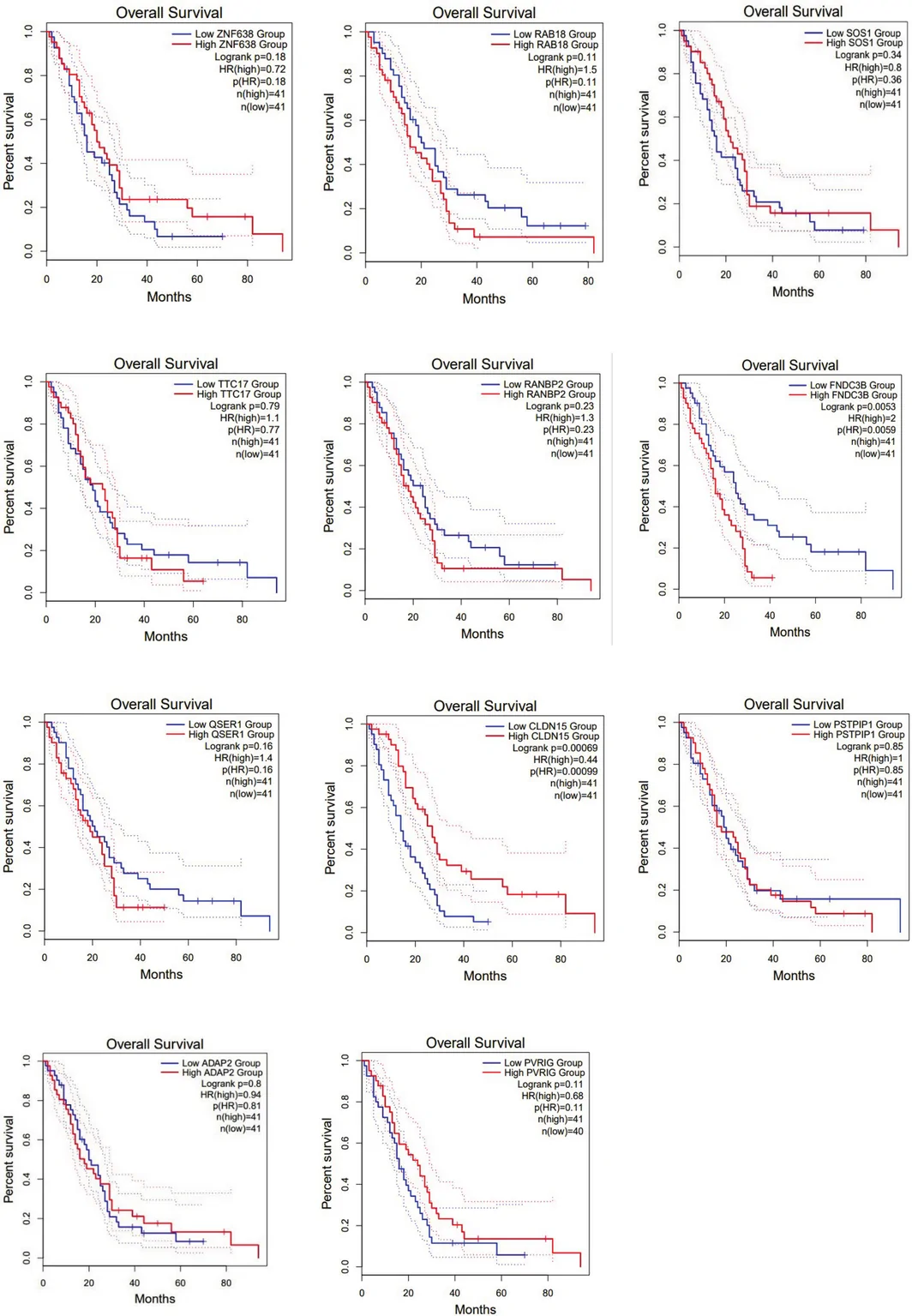
Survival curve for the hub genes (ZNF638, RAB18, SOS1, TTC17, RANBP2, FNDC3B, QSER1, CLDN15, PSTPIP1, ADAP2, and PVRIG).

### 3.6 Hub Genes Performance Assessment Using a Classification Algorithm

Figure 7 displays the classification performance of the hub‐gene panel, which was re‐evaluated using repeated stratified fivefold cross‐validation with 25 repeats. The performance of five popular machine learning classifiers was evaluated using their receiver operating characteristic (ROC) curves and corresponding area under the curve (AUC) values. The figure visually supports the performance metrics summarized in Table 2. The results show that the XGB model achieved the best performance, with the highest AUC of 97.53% (Figure 7). This indicates that the XGB model achieved high discriminatory performance in distinguishing between the two classes.

**FIGURE 7:**
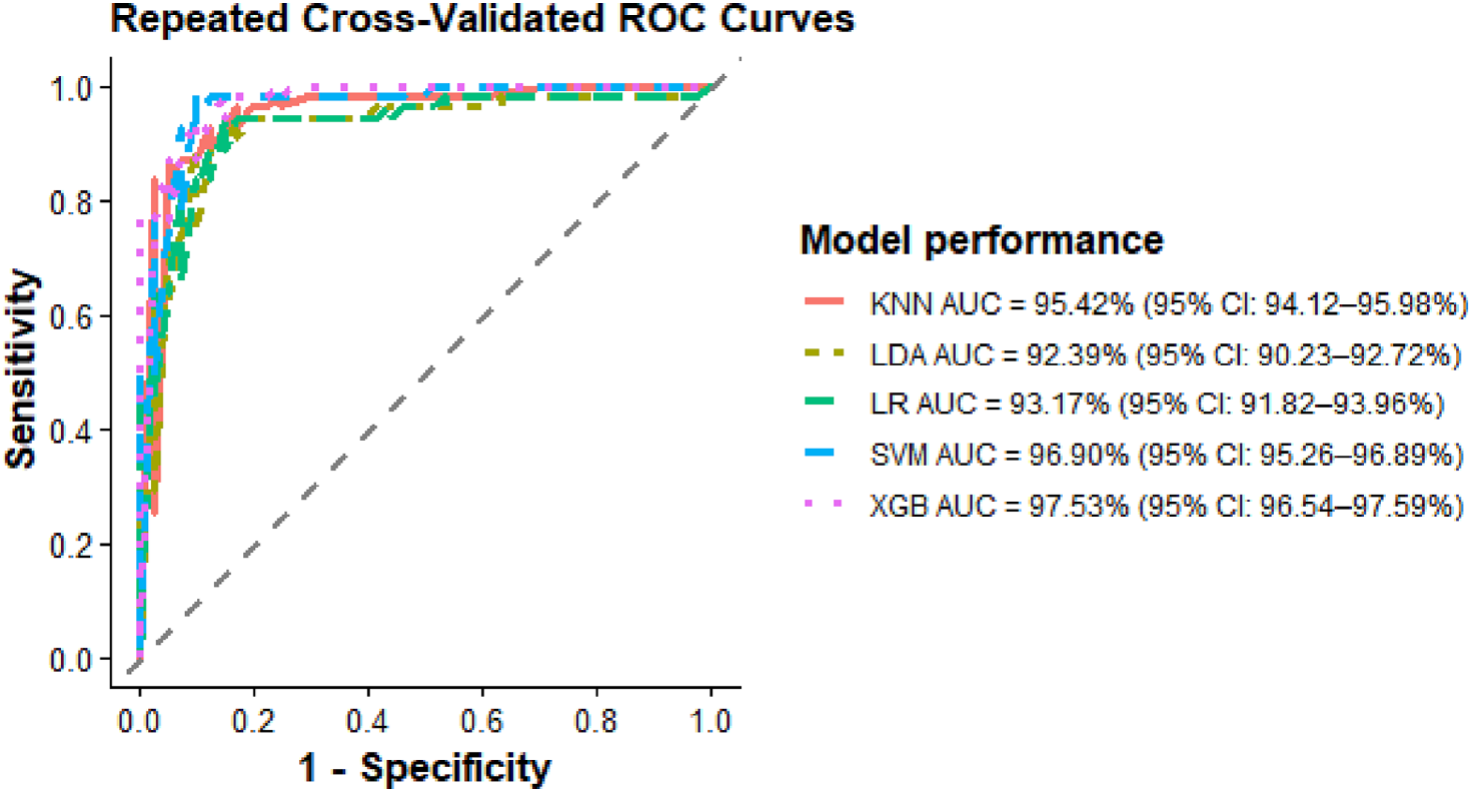
Repeated cross‐validated ROC curves for optimized machine learning classifiers.

**TABLE 2:** Classification performance (%) of machine‐learning models using the MPM hub‐gene panel.

Model	Accuracy (%)	AUC (%)	Pooled AUC 95% CI (%)	Sensitivity (%)	Specificity (%)
SVM	92.99 ± 5.45	96.90 ± 3.91	95.26–96.89	95.42 ± 6.07	89.77 ± 9.40
LDA	85.53 ± 7.62	92.39 ± 5.86	90.23–92.72	82.91 ± 12.94	89.09 ± 9.73
KNN	88.06 ± 7.64	95.42 ± 5.09	94.12–95.98	81.67 ± 12.8	96.63 ± 6.18
XGB	90.04 ± 5.94	97.53 ± 2.71	96.54–97.59	90.69 ± 8.83	89.12 ± 11.07
LR	88.66 ± 6.77	93.17 ± 5.22	91.82–93.96	91.27 ± 9.19	85.16 ± 11.13

The SVM model also performed exceptionally well, with the curve reaching an AUC‐Mean of 96.90%, placing it just below the XGBoost AUC‐Mean of 97.53%. The KNN model has an AUC‐Mean of 95.50%, indicating a strong but slightly lower performance. The LDA curves, with an AUC‐Mean of 92.39%, were significantly lower than those of the other models and suggest it was a less effective classifier for this dataset. This visual evidence from Figure 7 confirms the performance rankings detailed in the accompanying text and Table 2. The optimized hyperparameters of machine learning are in Table S4, and the mean performance is in Table S5.

## 4 Discussion

In MPM, the combination of immune pathway changes, genetic deregulation, and environmental exposure is especially important due to the intricate molecular exposure underlying the disease's evolution. Shared DEGs between tumor and normal tissues reveal the complex web of biological processes that are disrupted, including apoptosis, cell cycle regulation, and inflammation [28, 29]. These results highlight the value of integrative analysis in identifying possible treatment targets and biomarkers. It is crucial to comprehend the multifaceted nature of MPM, which is influenced by immunological modulation, gene co‐expression dynamics, and asbestos exposure to advance personalized treatment and enhance clinical results [30].

To identify the key genes and pathways in MPM, the current study used gene co‐expression network analysis. Between the Wilcoxon rank test and Limma package, we found 12 649 DEGs. Using module preservation analysis and WGCNA, five essential modules were identified in the GSE51024 mesothelioma dataset. For these crucial modules, GO and pathway enrichment analyses were performed. The machine‐learning analysis was conducted to assess whether the identified hub‐gene panel could distinguish MPM from normal samples in the available cohort. The 12 hub genes that we discovered in MMhub were thought to be the last potential molecular markers of MPM. These results might offer fresh perspectives on the pathophysiology of MPM.

Compared to other bioinformatics findings in MPM research [4, 11, 31], which are identified by differential analysis, the proposed method is quite different. For example, the gene co‐expression module analysis used in the study yields important gene modules rather than DEGs, offering systems biology insights. First, it grouped genes by condition and then identified hub genes, which are highly connected nodes within each network module. Joint analyses were conducted to precisely identify the hub genes from each module, then MM significance analysis was employed for choosing hub genes and uncovered common hub genes in MPM. Several machine‐learning classifiers were employed to evaluate the within‐cohort discriminatory performance of the identified hub‐gene panel between MPM tumor and paired normal lung samples. The performance of these advanced techniques was assessed, demonstrating high accuracy, AUC, specificity, and sensitivity.

This study offers fresh perspectives on the molecular causes of MPM by thoroughly examining the biological mechanisms and signaling pathways enriched in hub genes associated with the disease. MPM‐associated genes are primarily involved in essential biological processes, including cilium assembly, chromosomal segregation, mitotic nuclear division, and microtubule–based motility, according to the GO enrichment study. Malignant cells usually interfere with these activities, which are essential for preserving cytoskeletal integrity and controlling cell division [32]. Interestingly, changes in microtubule dynamics, a characteristic of cancer, have been connected to MPM's higher risk of metastasis and resistance to chemotherapy [33]. Additionally, the KEGG pathway analysis revealed notable enrichment in important signaling and cancer‐related pathways. Apoptosis and cell growth control were dysregulated in MPM, as evidenced by the marked enrichment of the cell cycle, cellular senescence, and FoxO signaling pathways [34]. Furthermore, cellular adhesion and intracellular transport processes may be crucial for the growth and metastasis of MPM, as shown by enrichment in endocytosis, focal adhesion, and tight junction pathways [35].

The study identified the Wnt signaling pathway and axon guidance, which play new roles in immune evasion and tumor microenvironment remodeling, and this finding is particularly intriguing [36]. These pathway interactions suggest a sophisticated system of regulatory circuits that may be used therapeutically. Taken as a whole, the results of this study highlighted the importance of the hub genes and pathways implicated in MPM pathogenesis. It offered a starting point for additional therapeutic targeting and functional confirmation. By combining GO and KEGG enrichment analysis, a deeper understanding of the molecular mechanisms underlying MPM can be gained.

Numerous bioinformatics projects have made extensive use of machine learning classifiers [9, 37, 38, 39]. To verify the correctness of the machine learning classification algorithms, they have been run on the MMhub gene data. It was found that the dataset of MPM tumor and normal tissue samples performed satisfactorily in sample categorization (accuracy > 0.90), thereby validating the hub genes identified by MM.

For understanding disease mechanisms and developing effective treatment plans, the discovery of hub genes (ZNF638, RAB18, SOS1, TTC17, RANBP2, FNDC3B, QSER1, CLDN15, PSTPIP1, SEPTIN6, ADAP2, and PVRIG) and shared genes associated with MPM has significant implications. These discovered hub genes might be desirable targets for drug development or predictors of disease course. Through H3K9me3 alteration, ZNF638 may function as an epigenetic repressor in MPM, perhaps silencing immune‐activating retroelements. By causing viral mimicry, its reduction increases susceptibility to immune checkpoint inhibitors (ICIs) by triggering innate immune responses and upregulating PD‐L1. ZNF638 targeting offers a novel approach to improving immunotherapy outcomes by increasing interferon signaling, triggering viral mimicry, and sensitizing MPM tumors to immune checkpoint therapy [40]. One of miR‐30b/c's direct functional targets in NSCLC cells was Rab18. In contrast to nearby nontumor tissues, nonsmall cell lung cancer (NSCLC) tissues also showed upregulation of Rab18 protein levels and downregulation of miR‐30b/c. Moreover, in vitro proliferation of NSCLC cells was inhibited by ectopic overexpression of miR‐30b/c [41]. Only three possible mutations were found among 810 tumor or tumor cell line samples screened for SOS1 mutations. The 95% confidence interval for SOS1 mutations in both the specific tumor types and all tumors was determined from the data. There may be a sizable number of SOS1 mutations in cancers for which fewer cases were examined, resulting in inadvertent undercounting. However, the total SOS1 mutation rate in human cancer is minimal [42].

When comparing ESCC cells, plasma, and tissues with normal samples, the expression level of circ‐TTC17 was noticeably higher. Circ‐TTC17 facilitated ESCC cell migration and proliferation in vitro. According to bioinformatics predictions, circ‐TTC17 may control the course of ESCC by serving as a microRNA sponge for microRNAs (miRNAs) [43]. Small cell lung cancer had substantially greater RANBP2 transcripts than NSCLC, despite the fact that RANBP2 mRNA expression was rarely downregulated in human lung cancer cell lines. The mRNA expression levels of the RANBP2, Topoll‐alpha, and Topoll‐beta genes did not correlate with the chemosensitivity of amrubicin [44].

A gene with an unclear function, glutamine and serine‐rich protein 1 (QSER1), is altered in several cancerous tissues [45]. High‐frequency mutations in the QSER1 gene may affect the clinical outcome of ovarian cancer treatment, according to studies on the disease [46]. It was discovered that nonsmall‐cell lung carcinoma had downregulated QSWR1 [47]. Furthermore, QSER1 was associated with HCC recurrence and expressed at significantly higher levels in HCC cells than in normal hepatocytes [48].

A study was conducted using MPM tissue samples, examined by immunohistochemistry, to determine whether CLD15 serves as a reliable positive marker for MPMs. Additionally, new rat antihuman claudin‐15 (CLDN15) monoclonal antibodies were created to recognize CLDN15 specifically [49].

Proline–serine–threonine phosphatase interacting protein 1 (PSTPIP1) is a cytoskeletal adaptor protein that is mostly produced in hematopoietic cells and is known to control immunological responses and actin cytoskeleton dynamics. Through interactions with pyrin that trigger the inflammasome and consequent IL‐1β production, mutations in PSTPIP1 have been linked to autoinflammatory diseases, including PAPA (pyogenic arthritis, pyoderma gangrenosum, and acne) syndrome [50].

Despite the paucity of direct research on PSTPIP1 in MPM, its role in regulating inflammatory pathways suggests its potential involvement in the tumor microenvironment. An important part of MPM pathophysiology is inflammation, which is frequently brought on by asbestos exposure and results in the release of cytokines like IL‐1β and persistent inflammatory responses. Because PSTPIP1 plays a role in cytokine modulation and inflammasome activation, abnormal PSTPIP1 expression or activity may be a factor in the pro‐inflammatory environment seen in MPM [51]. To clarify PSTPIP1's expression patterns in MPM tissues and its potential as a biomarker or therapeutic target, further investigation is necessary. Understanding PSTPIP's function may help uncover new treatment options and shed light on the inflammatory processes that worsen MPMs.

A number of the septin family of GTP‐binding cytoskeletal proteins, SEPTIN6 (septin), has been linked to important cellular functions such as vesicle trafficking and cytoskeletal remodeling. Given its role in regulating cell migration, proliferation, and apoptosis, its significance in cancer biology is increasingly recognized.

SEPTIN6 has been found to be overexpressed in tumor tissues of hepatocellular carcinoma (HCC) in contrast to nearby nontumor tissues. SEPTIN6 overexpression has been shown to stimulate HCC cell invasion, migration, and proliferation. SEPTIN6 has been shown to stimulate the production of F‐actin, which in turn dephosphorylates large tumor suppressor kinase 1 (LATS1), inhibits the Hippo signaling pathway, and activates yes‐associated protein (YAP). Because of this cascade, cyclin D1 and matrix metallopeptidase 2 (MMP2) have been upregulated, which has aided in the growth of tumors [52].

Despite the lack of concrete evidence in MPM, it has been proposed that SEPTIN6‐driven oncogenic pathways in other malignancies may also apply to MPM. Since SEPTIN6 maintains proper actin cytoskeletal architecture and cytokinesis, its dysregulation has been proposed as a potential factor in MPMs' abnormal cell division and invasive behavior [53].

Centaurin‐α 2, often referred to as ADAP2 (ArfGAP with dual PH domains 2), is a GTPase‐activating protein (GAP) that controls ARF6, a small GTPase involved in cell motility, membrane trafficking, and actin cytoskeleton remodeling. There have been reports of ADAP2 expression dysregulation in a number of pathological settings, such as inflammatory illnesses and several types of cancer.

ADAP2 overexpression has been linked to aggressive tumor activity in cancer biology. In particular, higher levels of ADAP2 mRNA and protein have been found in tumor tissues from clear cell renal cell carcinoma (ccRCC) compared with nearby normal tissues. Functional studies indicate that ADAP2 overexpression promotes the migration, invasion, and proliferation of ccRCC cells, whereas its knockdown prevents these effects. Furthermore, in patients with ccRCC, elevated ADAP2 expression has been linked to poor overall survival and progression‐free survival, indicating that it may be a useful prognostic indicator and therapeutic target for this cancer type [54].

While there are currently no direct studies on ADAP2 in MPM, its roles in membrane trafficking and cytoskeletal dynamics suggest it may affect tumor cell invasion and immune evasion in MPM. Further research on the expression patterns and functional roles of ADAP2 in MPM is necessary, given the disease's aggressive nature and its dependence on cellular mechanisms, including invasion and migration, for progression. Such research could determine whether changes in ADAP2 contribute to the pathophysiology of MPM and evaluate its potential as a biomarker or therapeutic target.

CD112R, often referred to as PVRIG (poliovirus receptor‐related immunoglobulin‐containing protein), is an inhibitory immunological checkpoint receptor that is expressed on T cells and natural killer (NK) cells. It has been demonstrated that its interaction with its receptor, CD112 (PVRL2), inhibits T cell activation and cytokine generation, which aids in immune evasion in several malignancies [55]. PVRIG's significance in other solid tumors implies possible relevance, despite the paucity of direct studies of the protein in MPM. Tumor‐infiltrating lymphocytes (TILs) in a variety of cancer types, including endometrial, ovarian, and lung malignancies, have been shown to express PVRIG, especially CD $8^{+}$ T cells with an exhausted phenotype. A complex network of immune control inside the tumor microenvironment is also indicated by the co‐expression of PVRIG with other inhibitory receptors, including PD‐1 and TIGIT, which has been documented [56]. PVRIG inhibition has been shown in preclinical research to improve T cell responses and slow tumor growth. In murine models, PVRIG was inhibited by antibody‐mediated suppression or genetic deletion, thereby enhancing CD8 T cell activation and working in concert with PD‐L1 blockade to further reduce tumor growth. These results imply that PVRIG targeting, either by itself or in conjunction with other checkpoint inhibitors, may provide therapeutic advantages for malignancies that exhibit immune suppression [57].

In summary, this study identified important molecular signatures in MPM by combining systems biology and machine learning. We identified conserved gene co‐expression networks using WGCNA and isolated and functionally annotated biologically significant hub genes from these modules. Gene ontology and pathway enrichment analyses identified important cellular functions, such as cilium organization and microtubule‐based motility, that may contribute to the progression of MPM. While classification models showed excellent predictive performance based on gene expression, the survival study further confirmed the prognostic significance of specific hub genes. These genes are computationally nominated candidates that require independent biological and clinical validation before diagnostic or therapeutic application.

### 4.1 Limitations

This study has several limitations. The analysis was based on a single public Affymetrix microarray dataset with a modest sample size, which limits the generalizability of the exploratory findings. Although RMA normalization was applied, complete batch‐level metadata were not available; therefore, residual batch effects or hidden technical confounders cannot be fully excluded. The comparator samples in GSE51024 were normal lung tissues rather than normal pleural mesothelial tissues. Module preservation should also be interpreted as an internal assessment of module stability rather than independent biological validation. In addition, important variables such as age, sex, histological subtype, smoking history, asbestos exposure, tumor stage, and treatment history were not fully available. Finally, the machine learning and biomarker findings are computational and require confirmation in independent MPM cohorts and through experimental validation, including qRT‐PCR, immunohistochemistry, and functional assays.

## 5 Conclusion

MPM is a highly aggressive cancer with limited treatment options and a dire prognosis. This study employed a multifaceted computational approach, integrating bioinformatics and machine learning, to uncover novel biomarkers and pathways critical to MPM pathogenesis. Using gene expression data from the GSE51024 dataset, we identified DEGs significantly altered in MPM tumor samples compared with normal lung tissue.

A key aspect of our methodology was the use of WGCNA to group genes into functional modules. This approach allowed us to identify 12 hub genes, including ZNF638, RAB18, SOS1, TTC17, RANBP2, FNDC3B, QSER1, CLDN15, PSTPIP1, SEPTIN6, ADAP2, and PVRIG, which are highly interconnected within these modules and are therefore likely to play a central role in the disease. Functional enrichment analyses indicate that these genes are associated with biological processes, including cell cycle regulation, immune response, and cancer progression.

Furthermore, we conducted survival analysis using GEPIA2, and the TCGA‐MESO cohort showed that 11 of the identified hub genes were associated with overall survival in mesothelioma. Higher expression of RAB18, TTC17, RANBP2, FNDC3B, and QSER1 was associated with poorer overall survival, suggesting their potential risk‐related prognostic relevance. In contrast, lower expression of ZNF638, CLDN15, and PVRIG was associated with poorer survival. In addition, to evaluate their potential discriminatory ability, we trained and tested various machine learning algorithms, which demonstrated high accuracy in distinguishing between tumor and normal samples. This integrative systems biology and machine learning approach provides useful insights into MPM‐associated molecular mechanisms and identifies candidate biomarkers with diagnostic and prognostic potential.

The identified genes provide potential insights into MPM‐associated molecular mechanisms and may serve as candidates for further investigation toward biomarker development and therapeutic research. However, future research should validate these findings using independent, larger patient cohorts and incorporate data from diverse clinical stages and treatment histories to ensure broader applicability. Future work should focus on in vitro and in vivo validation of the identified hub genes and their associated pathways to confirm their functional roles and explore their potential as therapeutic targets for drug development.

## Author Contributions

**Md. Lotifur Rohman:** conceptualization, writing – original draft, methodology, visualization, software, formal analysis, data curation, validation. **Suman Biswas:** conceptualization, writing – original draft, methodology, validation, visualization, writing – review and editing, software, data curation, formal analysis, supervision.

## Funding

The authors have nothing to report.

## Conflicts of Interest

The authors declare no conflicts of interest.

## Supporting information


**Table S1:** Sample label mapping.
**Table S2:** KEGG enrichment analysis.
**Table S3:** Combined annotated hub genes.
**Table S4:** Optimized hyperparameters.
**Table S5:** Mean performance.

## Data Availability

The data supporting the findings of this study are publicly available in the NCBI Gene Expression Omnibus (GEO) under accession number GSE51024 at https://www.ncbi.nlm.nih.gov/geo/query/acc.cgi?acc=GSE51024.
