# Supplementary material for: Integrative Bioinformatics and Machine Learning Approaches for Biomarker and Pathway Discovery in Malignant Pleural Mesothelioma
Source: Cancer Rep (Hoboken). 2026 Oct 26;9(10):e70731. doi: 10.1002/cnr2.70731 (PMC13697528; doi:10.1002/cnr2.70731)
Supplement: Supplementary file 1 [file CNR2-9-e70731-s001.docx]

**Table S1.** Sample Label Mapping

| **Original Sample** | **New Label** | **Group** |  | **Original Sample (Cont.)** | **New Label** | **Group** |
| --- | --- | --- | --- | --- | --- | --- |
| GSM1235016_Normal1.CEL.gz | Normal1 | Normal |  | GSM1235064_Normal24.CEL.gz | Normal24 | Normal |
| GSM1235017_Tumor1.CEL.gz | Tumor1 | Tumor |  | GSM1235065_Tumor24.CEL.gz | Tumor26 | Tumor |
| GSM1235018_Normal2.CEL.gz | Normal2 | Normal |  | GSM1235066_Normal25.CEL.gz | Normal25 | Normal |
| GSM1235019_Tumor2.CEL.gz | Tumor2 | Tumor |  | GSM1235067_Tumor25.CEL.gz | Tumor27 | Tumor |
| GSM1235020_Normal3.CEL.gz | Normal3 | Normal |  | GSM1235068_Normal26.CEL.gz | Normal26 | Normal |
| GSM1235021_Tumor3.CEL.gz | Tumor3 | Tumor |  | GSM1235069_Tumor26.CEL.gz | Tumor28 | Tumor |
| GSM1235022_Normal4.CEL.gz | Normal4 | Normal |  | GSM1235070_Normal27.CEL.gz | Normal27 | Normal |
| GSM1235023_Tumor4.CEL.gz | Tumor4 | Tumor |  | GSM1235071_Tumor27.CEL.gz | Tumor29 | Tumor |
| GSM1235024_Normal5.CEL.gz | Normal5 | Normal |  | GSM1235072_Normal28.CEL.gz | Normal28 | Normal |
| GSM1235025_Tumor5.CEL.gz | Tumor5 | Tumor |  | GSM1235073_Tumor28.CEL.gz | Tumor30 | Tumor |
| GSM1235026_Normal6.CEL.gz | Normal6 | Normal |  | GSM1235074_Tumor54.CEL.gz | Tumor31 | Tumor |
| GSM1235027_Tumor6.CEL.gz | Tumor6 | Tumor |  | GSM1235075_Tumor55.CEL.gz | Tumor32 | Tumor |
| GSM1235028_Normal7.CEL.gz | Normal7 | Normal |  | GSM1235076_Normal29.CEL.gz | Normal29 | Normal |
| GSM1235029_Tumor7.CEL.gz | Tumor7 | Tumor |  | GSM1235077_Tumor29.CEL.gz | Tumor33 | Tumor |
| GSM1235030_Normal8.CEL.gz | Normal8 | Normal |  | GSM1235078_Normal30.CEL.gz | Normal30 | Normal |
| GSM1235031_Tumor8.CEL.gz | Tumor8 | Tumor |  | GSM1235079_Tumor30.CEL.gz | Tumor34 | Tumor |
| GSM1235032_Normal9.CEL.gz | Normal9 | Normal |  | GSM1235080_Normal31.CEL.gz | Normal31 | Normal |
| GSM1235033_Tumor9.CEL.gz | Tumor9 | Tumor |  | GSM1235081_Tumor31.CEL.gz | Tumor35 | Tumor |
| GSM1235034_Normal10.CEL.gz | Normal10 | Normal |  | GSM1235082_Normal32.CEL.gz | Normal32 | Normal |
| GSM1235035_Tumor10.CEL.gz | Tumor10 | Tumor |  | GSM1235083_Tumor32.CEL.gz | Tumor36 | Tumor |
| GSM1235036_Normal11.CEL.gz | Normal11 | Normal |  | GSM1235084_Tumor42.CEL.gz | Tumor37 | Tumor |
| GSM1235037_Tumor11.CEL.gz | Tumor11 | Tumor |  | GSM1235085_Tumor43.CEL.gz | Tumor38 | Tumor |
| GSM1235038_Normal12.CEL.gz | Normal12 | Normal |  | GSM1235086_Normal33.CEL.gz | Normal33 | Normal |
| GSM1235039_Tumor12.CEL.gz | Tumor12 | Tumor |  | GSM1235087_Tumor33.CEL.gz | Tumor39 | Tumor |
| GSM1235040_Normal13.CEL.gz | Normal13 | Normal |  | GSM1235088_Normal34.CEL.gz | Normal34 | Normal |
| GSM1235041_Tumor13.CEL.gz | Tumor13 | Tumor |  | GSM1235089_Tumor34.CEL.gz | Tumor40 | Tumor |
| GSM1235042_Normal14.CEL.gz | Normal14 | Normal |  | GSM1235090_Normal35.CEL.gz | Normal35 | Normal |
| GSM1235043_Tumor14.CEL.gz | Tumor14 | Tumor |  | GSM1235091_Tumor35.CEL.gz | Tumor41 | Tumor |
| GSM1235044_Normal15.CEL.gz | Normal15 | Normal |  | GSM1235092_Tumor44.CEL.gz | Tumor42 | Tumor |
| GSM1235045_Tumor15.CEL.gz | Tumor15 | Tumor |  | GSM1235093_Tumor45.CEL.gz | Tumor43 | Tumor |
| GSM1235046_Normal16.CEL.gz | Normal16 | Normal |  | GSM1235094_Tumor46.CEL.gz | Tumor44 | Tumor |
| GSM1235047_Tumor16.CEL.gz | Tumor16 | Tumor |  | GSM1235095_Tumor47.CEL.gz | Tumor45 | Tumor |
| GSM1235048_Normal17.CEL.gz | Normal17 | Normal |  | GSM1235096_Tumor48.CEL.gz | Tumor46 | Tumor |
| GSM1235049_Tumor17.CEL.gz | Tumor17 | Tumor |  | GSM1235097_Tumor49.CEL.gz | Tumor47 | Tumor |
| GSM1235050_Normal18.CEL.gz | Normal18 | Normal |  | GSM1235098_Tumor50.CEL.gz | Tumor48 | Tumor |
| GSM1235051_Tumor18.CEL.gz | Tumor18 | Tumor |  | GSM1235099_Tumor51.CEL.gz | Tumor49 | Tumor |
| GSM1235052_Normal19.CEL.gz | Normal19 | Normal |  | GSM1235100_Normal36.CEL.gz | Normal36 | Normal |
| GSM1235053_Tumor19.CEL.gz | Tumor19 | Tumor |  | GSM1235101_Tumor36.CEL.gz | Tumor50 | Tumor |
| GSM1235054_Normal20.CEL.gz | Normal20 | Normal |  | GSM1235102_Normal37.CEL.gz | Normal37 | Normal |
| GSM1235055_Tumor20.CEL.gz | Tumor20 | Tumor |  | GSM1235103_Tumor37.CEL.gz | Tumor51 | Tumor |
| GSM1235056_Normal21.CEL.gz | Normal21 | Normal |  | GSM1235104_Normal38.CEL.gz | Normal38 | Normal |
| GSM1235057_Tumor21.CEL.gz | Tumor21 | Tumor |  | GSM1235105_Tumor38.CEL.gz | Tumor52 | Tumor |
| GSM1235058_Normal22.CEL.gz | Normal22 | Normal |  | GSM1235106_Normal39.CEL.gz | Normal39 | Normal |
| GSM1235059_Tumor22.CEL.gz | Tumor22 | Tumor |  | GSM1235107_Tumor39.CEL.gz | Tumor53 | Tumor |
| GSM1235060_Normal23.CEL.gz | Normal23 | Normal |  | GSM1235108_Normal40.CEL.gz | Normal40 | Normal |
| GSM1235061_Tumor23.CEL.gz | Tumor23 | Tumor |  | GSM1235109_Tumor40.CEL.gz | Tumor54 | Tumor |
| GSM1235062_Tumor52.CEL.gz | Tumor24 | Tumor |  | GSM1235110_Normal41.CEL.gz | Normal41 | Normal |
| GSM1235063_Tumor53.CEL.gz | Tumor25 | Tumor |  | GSM1235111_Tumor41.CEL.gz | Tumor55 | Tumor |

**Table S2.** KEGG Enrichment Analysis

| **category** | **subcategory** | **ID** | **Description** | **GeneRatio** | **BgRatio** | **RichFactor** | **Fold Enrichment** | **zScore** | **pvalue** | **p.adjust** | **qvalue** | **geneID** | **Count** |
| --- | --- | --- | --- | --- | --- | --- | --- | --- | --- | --- | --- | --- | --- |
| Cellular Processes | Cell growth and death | hsa04110 | Cell cycle | 98/3200 | 158/8541 | 0.620253165 | 1.6555 | 6.4371 | 0.0000 | 0.0000 | 0.0000 | 27085/57082/151648/5527/4085/8243/993/9126/472/5885/6500/894/7533/5111/5516/9184/896/7157/4174/4175/4171/5525/5347/1019/10274/4089/9133/991/4087/983/890/9232/6502/1871/701/990/10912/8318/1031/10403/10926/23047/9700/7272/9134/4998/995/8556/5933/1111/4088/8900/1029/4616/8454/1663/595/4176/546/25847/2810/9212/699/7043/81620/545/1032/11200/5001/1022/4193/4173/5934/1028/898/25836/891/23383/996/64682/23594/51529/119504/113130/1021/246184/51434/2932/5529/1870/90381/157570/114799/5515/51433/1030/80174/10393 | 98 |
| Cellular Processes | Cell growth and death | hsa04218 | Cellular senescence | 89/3200 | 157/8541 | 0.566878981 | 1.5130 | 5.0219 | 0.0000 | 0.0001 | 0.0001 | 5594/204851/993/776/5296/472/801/894/3710/678/5500/9261/896/4605/7157/1019/5605/5530/2305/4893/3709/9133/2308/8503/291/3576/4087/983/890/1871/3708/5293/10912/2309/9134/5933/1111/4361/4088/2626/8900/7046/5533/1029/207/805/4775/4616/5606/3552/10111/824/595/7048/4790/23291/7043/545/5603/3486/11200/5600/4193/677/5934/6237/8878/898/55957/891/7249/8945/4683/5291/23411/1978/10000/54822/1021/28996/10114/1870/5810/286826/823/1030/208/5928/6009 | 89 |
| Cellular Processes | Transport and catabolism | hsa04144 | Endocytosis | 129/3200 | 252/8541 | 0.511904762 | 1.3663 | 4.5686 | 0.0000 | 0.0005 | 0.0004 | 3310/55680/51699/5156/387/30844/5337/2869/10097/55048/157/119016/8724/8766/10552/5878/1601/3482/6642/7251/9559/6455/10095/3799/3949/1175/5590/26286/7456/857/4087/382/858/409/22905/5867/2263/9922/2261/2348/2264/2350/8411/9372/273/4088/8395/6457/6456/3559/5371/7046/1234/3579/868/829/998/7048/51100/9266/5584/83737/56288/3305/253725/4193/84364/387680/23327/23527/23396/23111/11059/57154/116984/9135/1759/10564/58533/10093/51652/55738/28964/51510/57132/29082/56904/408/64411/27128/80223/23624/81873/89853/79720/30846/58513/55737/10015/51028/10890/27131/653857/50807/9265/112936/128866/116983/9815/84440/92421/84552/10617/93343/10254/9727/867/30845/147179/6643/29934/9146/84612/30011/100287171/10096/1213/642517/9765 | 129 |
| Cellular Processes | Cellular community - eukaryotes | hsa04510 | Focal adhesion | 105/3200 | 203/8541 | 0.517241379 | 1.3805 | 4.2474 | 0.0000 | 0.0014 | 0.0011 | 5594/256076/22801/5156/387/10627/1277/6654/2335/3672/6696/5296/3915/894/10398/5829/3693/3611/5500/1293/3725/6464/4659/896/4660/1398/5906/1278/8503/857/7058/858/2002/3909/5293/5155/5170/7448/3690/1299/7060/5602/2013/3691/3673/3908/8395/1311/3696/2277/8515/207/3694/2317/87/824/595/998/5062/1292/3914/3479/7059/5228/7422/5923/3082/1291/23396/83660/6655/5578/4233/7143/8516/1286/1285/2885/3688/2064/1280/5291/10451/80310/10319/103910/2321/10000/5601/2932/2889/673/5058/284217/3680/53358/2324/330/131873/1793/55742/394/3381/208/5747 | 105 |
| Human Diseases | Cancer: specific types | hsa05212 | Pancreatic cancer | 47/3200 | 77/8541 | 0.61038961 | 1.6292 | 4.2926 | 0.0000 | 0.0014 | 0.0011 | 5594/51426/5337/5296/7157/369/5899/1019/4089/8503/10928/4087/1643/1871/578/6199/5293/10912/5602/7039/5888/4088/598/7046/1029/207/4616/675/595/998/7048/6774/5900/4790/9459/7043/6772/7422/2064/5291/10000/1021/5898/5601/673/1870/208 | 47 |
| Cellular Processes | Cell motility | hsa04814 | Motor proteins | 102/3200 | 197/8541 | 0.517766497 | 1.3820 | 4.1980 | 0.0000 | 0.0014 | 0.0011 | 221458/1768/8701/124602/200132/4651/79819/10627/84516/7168/55567/7139/7171/127602/4629/53904/10121/10398/3799/6993/10120/3835/72/23303/4646/10671/6990/22920/3797/1783/51626/10126/7280/11258/3832/4644/9493/1062/7802/9585/1780/9928/1770/4647/829/10383/203068/7846/10749/11004/3801/3833/7134/51174/7277/4430/4628/3831/347733/56171/4650/92558/79784/24137/10112/55930/4649/56992/80179/55614/55083/27019/64446/81930/103910/146909/57576/9371/4645/140735/55605/51175/83544/90990/57519/25981/64147/11127/196385/81027/1767/55582/1769/146439/1781/7170/83657/146754/126820/201625/1778/4641 | 102 |
| Environmental Information Processing | Signal transduction | hsa04068 | FoxO signaling pathway | 73/3200 | 133/8541 | 0.54887218 | 1.4650 | 4.1831 | 0.0000 | 0.0014 | 0.0011 | 5594/10018/9455/6654/5296/472/894/847/5564/369/10769/5347/1454/5605/4089/4893/8743/9133/2308/8503/604/6502/5293/10912/2309/5170/1901/9454/3667/5602/9140/10733/4088/4303/3575/7046/207/3586/4616/595/23710/7048/6774/11345/11067/3479/7043/5603/1032/5600/4193/5934/6655/9456/891/2885/1263/5291/23411/23678/92579/7874/10000/6789/114907/5601/5562/673/5563/1030/208/51701/100132074 | 73 |
| Cellular Processes | Cellular community - eukaryotes | hsa04530 | Tight junction | 88/3200 | 170/8541 | 0.517647059 | 1.3816 | 3.8902 | 0.0001 | 0.0038 | 0.0029 | 24146/3993/8189/387/79778/10627/776/10097/4629/10552/10398/5111/9368/5516/1364/3725/5564/10095/7082/9414/5590/1019/5872/5906/1739/1366/11346/51421/9693/1365/7122/5602/5962/1080/8777/9071/2626/5581/9181/7430/87/595/998/3308/7846/5584/100506658/56288/7277/4628/23327/27134/2017/51208/9073/3688/2064/79784/10093/55114/58494/54566/81873/103910/50848/55844/57530/5522/10207/653857/4301/154810/9223/5601/84952/5562/64398/84552/5563/5520/137075/92359/153562/84612/5515/3996/91862/10096 | 88 |
| Organismal Systems | Development and regeneration | hsa04360 | Axon guidance | 94/3200 | 184/8541 | 0.510869565 | 1.3635 | 3.8586 | 0.0001 | 0.0038 | 0.0029 | 5594/55740/57522/387/10627/23380/5336/91584/5296/1808/3983/2771/10398/3611/23365/1942/5590/3985/5530/4893/8503/8440/10507/55558/6387/10512/6586/5998/5293/1020/3984/5170/3897/2049/2051/1945/22885/7220/2041/7225/9723/5533/5362/4775/998/5062/2048/29984/5781/27289/56288/10505/6237/818/5578/219699/4233/1946/3688/5291/6608/64221/56920/103910/5361/59277/57556/56963/64101/10501/91653/1073/2932/80031/5058/6092/84552/25791/8633/84448/817/2045/2043/658/9353/659/10509/84612/90249/4690/9860/1630/1943/5747 | 94 |
| Environmental Information Processing | Signal transduction | hsa04012 | ErbB signaling pathway | 49/3200 | 86/8541 | 0.569767442 | 1.5207 | 3.7567 | 0.0002 | 0.0060 | 0.0046 | 5594/374/6654/5336/5296/3725/6464/369/1398/5605/2065/4893/8503/6776/8440/2002/6199/1839/5293/5602/7039/3084/2066/207/685/868/5062/818/6655/5578/2885/2064/5291/1978/10000/5601/2549/2932/673/5058/27/817/867/10718/53358/208/4690/5747/145957 | 49 |
| Human Diseases | Cancer: specific types | hsa05220 | Chronic myeloid leukemia | 44/3200 | 77/8541 | 0.571428571 | 1.5252 | 3.5831 | 0.0003 | 0.0092 | 0.0070 | 5594/51426/6654/5296/1488/6464/4792/7157/369/1398/1019/5605/4089/4893/8503/6776/1643/1871/578/5293/10912/4088/598/7046/1029/207/4616/595/7048/4790/7043/5781/4193/6655/2885/5291/2122/10000/1021/673/1870/867/53358/208 | 44 |
| Cellular Processes | Transport and catabolism | hsa04148 | Efferocytosis | 80/3200 | 157/8541 | 0.50955414 | 1.3600 | 3.5242 | 0.0003 | 0.0092 | 0.0070 | 5594/5743/23166/84868/54896/286046/6566/5175/23355/23200/8754/19/4035/4953/51552/3693/5878/6513/9261/1398/5605/6256/102/140885/1374/6376/384/51454/240/23659/9844/65082/3690/1901/1844/9392/383/5029/948/5732/9023/8578/840/246/4775/3586/5031/5468/100133941/682/5781/5603/177/405/834/5600/23339/488/818/811/161291/23250/1849/63982/7376/1788/55113/23411/64284/51761/55423/80824/961/286410/126129/817/338382/1793/5087/5747 | 80 |
| Human Diseases | Endocrine and metabolic disease | hsa04933 | AGE-RAGE signaling pathway in diabetic complications | 55/3200 | 101/8541 | 0.544554455 | 1.4534 | 3.5482 | 0.0004 | 0.0092 | 0.0070 | 5594/1277/5336/2335/1906/5296/3725/1281/5590/1019/1278/4089/5580/3383/4893/2308/8503/3576/6776/4087/7412/5293/7056/5332/2152/5602/51196/4088/5581/2277/7046/207/3552/595/998/7048/6774/4790/7043/6772/5603/177/7422/5600/5578/5331/1286/1285/5291/50507/10000/5601/1958/113026/208 | 55 |
| Human Diseases | Cancer: specific types | hsa05223 | Non-small cell lung cancer | 42/3200 | 73/8541 | 0.575342466 | 1.5356 | 3.5573 | 0.0004 | 0.0092 | 0.0070 | 5594/51426/6654/5336/5296/7157/369/3799/1019/5605/6256/4893/8503/6776/1643/1871/578/5293/10912/2309/5170/7039/5979/1029/207/4616/238/595/6774/3082/6655/5578/4233/2885/2064/5291/10000/6789/1021/673/1870/208 | 42 |
| Human Diseases | Cancer: overview | hsa05205 | Proteoglycans in cancer | 100/3200 | 204/8541 | 0.490196078 | 1.3084 | 3.4504 | 0.0004 | 0.0099 | 0.0076 | 5594/387/1277/6654/5336/2335/5296/5829/3693/7078/3710/6382/23365/5500/4659/3339/7157/369/4660/6385/1278/3481/5605/2065/4893/3709/8503/2817/857/4087/858/2002/3708/6199/1839/5293/4318/5170/7448/3690/5962/3673/51196/2099/5328/7472/7855/288/7482/2066/207/7430/595/998/6774/2719/3479/5781/5603/2535/7422/8826/2260/3082/5600/4193/27250/6237/818/6655/5578/4233/7291/2017/5329/2885/3688/287/8325/2064/5291/29102/6608/10451/10855/79923/7099/7473/10000/80326/1655/2549/673/5058/817/867/3549/117581/208/5747 | 100 |
| Human Diseases | Cancer: specific types | hsa05210 | Colorectal cancer | 48/3200 | 87/8541 | 0.551724138 | 1.4726 | 3.4293 | 0.0006 | 0.0120 | 0.0092 | 5594/10018/51426/387/332/374/6654/5296/3725/7157/369/5899/5605/4089/4893/8503/4087/1643/578/6199/5293/10912/5366/5602/7039/4088/7046/207/4616/595/7048/5900/7043/51176/6655/2885/324/5291/83439/8313/10000/5898/5601/2932/673/54205/208/1630 | 48 |
| Cellular Processes | Cell growth and death | hsa04115 | p53 signaling pathway | 42/3200 | 75/8541 | 0.56 | 1.4947 | 3.3305 | 0.0008 | 0.0163 | 0.0125 | 841/64393/472/894/896/7157/6241/1019/9133/983/1643/3732/10912/5366/51512/637/9134/1111/598/1029/4616/595/2810/8795/3479/545/3486/11200/9540/4193/898/891/7249/64065/27244/55367/56475/83667/84883/1021/54205/8493 | 42 |
| Human Diseases | Drug resistance: antineoplastic | hsa01521 | EGFR tyrosine kinase inhibitor resistance | 44/3200 | 80/8541 | 0.55 | 1.4680 | 3.2551 | 0.0010 | 0.0189 | 0.0146 | 5594/10018/5156/6654/5336/5296/1977/6464/369/5605/2065/4893/8503/2263/6199/5293/2309/5155/4763/2261/7039/3570/3084/598/207/6774/3479/7422/3082/6655/5578/9470/4233/2885/2064/5291/80310/1978/10000/2549/2932/673/53358/208 | 44 |
| Environmental Information Processing | Signaling molecules and interaction | hsa04512 | ECM-receptor interaction | 48/3200 | 89/8541 | 0.539325843 | 1.4395 | 3.2260 | 0.0011 | 0.0189 | 0.0146 | 256076/22801/1277/2335/3672/6696/3915/3693/6382/1293/3339/6385/1278/7058/3909/7448/3690/1299/7060/3691/3673/3908/1311/3696/948/8515/3161/2811/3694/1292/3914/7059/1291/375790/7143/8516/1286/1285/3688/1280/10319/255743/961/284217/3680/131873/341640/3381 | 48 |
| Human Diseases | Cancer: specific types | hsa05215 | Prostate cancer | 52/3200 | 98/8541 | 0.530612245 | 1.4162 | 3.2077 | 0.0011 | 0.0189 | 0.0146 | 5594/5156/6654/5296/7113/7184/468/2950/4792/7157/369/5605/4893/2308/8503/6692/2263/1871/5293/4318/5155/5170/7039/9134/7850/5328/4314/6716/207/595/4790/3479/51176/2260/367/2078/4193/6655/898/3326/2885/2064/5291/80310/83439/10000/2932/673/1870/64764/208/6935 | 52 |
| Human Diseases | Cancer: specific types | hsa05214 | Glioma | 42/3200 | 76/8541 | 0.552631579 | 1.4750 | 3.2195 | 0.0012 | 0.0189 | 0.0146 | 5594/5156/51426/6654/5336/8536/5296/801/6464/7157/369/1019/5605/4893/8503/1643/1871/578/5293/10912/5155/7039/1029/207/805/4616/595/3479/4193/818/6655/5578/2885/5291/10000/1021/57118/673/1870/817/53358/208 | 42 |
| Human Diseases | Cancer: specific types | hsa05213 | Endometrial cancer | 34/3200 | 59/8541 | 0.576271186 | 1.5381 | 3.2102 | 0.0013 | 0.0198 | 0.0152 | 5594/51426/6654/1495/5296/999/3611/7157/369/5605/4893/8503/1643/2002/578/5293/10912/2309/5170/207/4616/595/51176/6655/2885/324/2064/5291/83439/8313/10000/2932/673/208 | 34 |
| Human Diseases | Cancer: specific types | hsa05211 | Renal cell carcinoma | 39/3200 | 70/8541 | 0.557142857 | 1.4870 | 3.1670 | 0.0014 | 0.0201 | 0.0155 | 5594/6654/5296/6923/2034/6513/3725/369/1398/5906/5605/4893/8503/9915/7428/5293/5155/7039/207/998/5062/7043/5781/7422/405/3082/6655/4233/2885/5291/112399/112398/54583/10000/2549/2889/673/5058/208 | 39 |
| Genetic Information Processing | Replication and repair | hsa03460 | Fanconi anemia pathway | 32/3200 | 55/8541 | 0.581818182 | 1.5529 | 3.1840 | 0.0014 | 0.0201 | 0.0155 | 2187/57697/51426/197342/5429/3280/2189/2175/672/5888/641/675/201254/9894/5395/545/55215/8940/91442/79008/55120/2188/79728/83990/80233/29089/116028/146956/22909/2177/353497/51455 | 32 |
| Human Diseases | Cancer: specific types | hsa05221 | Acute myeloid leukemia | 38/3200 | 68/8541 | 0.558823529 | 1.4915 | 3.1498 | 0.0015 | 0.0204 | 0.0157 | 5594/6654/862/5296/3728/929/369/5605/4893/8503/6776/1436/890/5914/6199/5293/1050/3815/8864/597/7704/8900/5371/207/595/1848/6774/4790/51176/6655/2885/2209/5291/83439/1978/10000/673/208 | 38 |
| Environmental Information Processing | Signal transduction | hsa04151 | PI3K-Akt signaling pathway | 163/3200 | 362/8541 | 0.450276243 | 1.2018 | 3.0370 | 0.0016 | 0.0208 | 0.0160 | 5594/10018/1441/256076/5527/22801/5156/2252/1386/2786/1277/374/6654/2335/3672/27006/6696/5296/7184/2782/3915/468/4170/894/7533/3693/5516/1977/1293/896/7157/1942/5525/1019/3164/1278/3481/5605/6256/2065/4893/8503/7058/1436/3566/2263/3909/6199/5293/10681/2791/2309/5155/2261/5170/672/7448/2264/3690/3667/1299/7060/4602/3691/7039/3673/9134/3815/1945/3908/2246/284/1311/3575/3696/5979/3570/3559/5294/627/2254/598/7010/2277/8515/2066/4803/2846/8817/2787/207/1440/3694/595/1292/4790/3914/3479/7059/5228/9863/7422/2260/3082/4193/1291/5934/6655/5578/898/9470/4233/7143/1946/8516/3326/1286/1285/2885/7249/3688/2064/1280/5291/55012/80310/10319/23678/64223/7099/5623/1978/92579/55844/4915/2321/10000/59345/5522/1021/9223/5562/2932/4254/284217/3680/5529/5563/5520/64764/2788/2324/57121/131873/23566/4909/5515/3381/208/1943/5747/6009/117145 | 163 |
| Environmental Information Processing | Signal transduction | hsa04390 | Hippo signaling pathway | 77/3200 | 157/8541 | 0.49044586 | 1.3090 | 3.0250 | 0.0018 | 0.0236 | 0.0181 | 286204/3993/332/374/1495/894/7533/999/5516/5500/3398/896/1857/25937/5590/1454/1739/4089/4087/7159/4092/2246/650/4088/653/7472/7855/654/7482/268/7046/2736/595/1453/3397/7048/23291/5584/7043/56288/2535/51176/652/6591/6657/324/8945/8325/60485/8994/353500/83439/7473/55844/8313/5522/10207/80326/10413/122786/64398/2932/84552/126374/5520/166824/85407/658/330/659/85409/84612/5515/3996/23286/84962/1856 | 77 |
| Organismal Systems | Endocrine system | hsa04917 | Prolactin signaling pathway | 39/3200 | 71/8541 | 0.549295775 | 1.4661 | 3.0526 | 0.0020 | 0.0239 | 0.0184 | 5594/6654/5296/894/6464/5605/3659/4893/8503/6776/2592/8835/5293/2309/5602/2099/9021/8792/207/595/6774/4790/9655/6772/5603/8600/5600/6655/2885/5291/2001/1154/10000/5601/122809/2932/9306/53358/208 | 39 |
| Genetic Information Processing | Folding, sorting and degradation | hsa04130 | SNARE interactions in vesicular transport | 21/3200 | 33/8541 | 0.636363636 | 1.6985 | 3.1117 | 0.0020 | 0.0239 | 0.0184 | 143187/415117/9341/6844/8673/8417/6804/6843/2054/8773/9554/6809/9570/8676/10228/9527/8674/10652/51272/10490/6810 | 21 |
| Environmental Information Processing | Signal transduction | hsa04340 | Hedgehog signaling pathway | 32/3200 | 56/8541 | 0.571428571 | 1.5252 | 3.0519 | 0.0021 | 0.0242 | 0.0186 | 157/894/8452/1454/1455/409/23288/2619/8405/2736/8454/595/1453/23291/57154/8945/6608/408/1456/53944/51684/91653/339745/2932/730094/2737/50937/11127/3549/132884/64399/23432 | 32 |
| Environmental Information Processing | Signal transduction | hsa04152 | AMPK signaling pathway | 61/3200 | 122/8541 | 0.5 | 1.3345 | 2.8806 | 0.0030 | 0.0331 | 0.0255 | 5527/84335/5296/51552/79602/5516/5564/5525/5209/2308/8503/890/1374/6199/5293/2309/5170/3667/1080/8900/948/207/3953/5468/595/5862/3479/2203/5208/23216/10645/31/7249/5291/51094/51719/55012/23417/23411/10891/79966/81617/1978/92579/55844/10000/10890/55437/5522/5562/126129/5529/5563/5520/5210/64764/5515/148/208/6009/92335 | 61 |
| Cellular Processes | Cell growth and death | hsa04210 | Apoptosis | 67/3200 | 136/8541 | 0.492647059 | 1.3149 | 2.8653 | 0.0031 | 0.0331 | 0.0255 | 5594/10018/841/4000/332/100506742/5296/472/468/4170/3710/3725/4792/7157/1512/5605/1513/8772/4893/3709/8743/8503/1520/4001/3708/578/1519/5293/10912/5783/5366/637/5170/5602/9020/7185/597/598/1677/4803/207/840/4616/8837/142/824/7846/4790/8795/1515/5414/27429/839/7277/1508/5551/6709/84823/5291/55367/10000/153090/5601/54205/330/823/208 | 67 |
| Organismal Systems | Environmental adaptation | hsa04710 | Circadian rhythm | 21/3200 | 34/8541 | 0.617647059 | 1.6485 | 2.9328 | 0.0034 | 0.0357 | 0.0275 | 8863/6500/5564/1454/5187/4783/9572/8864/8454/1453/23291/1407/6095/1408/4862/8945/79365/26224/5562/5563/6097 | 21 |
| Cellular Processes | Cellular community - eukaryotes | hsa04520 | Adherens junction | 48/3200 | 93/8541 | 0.516129032 | 1.3776 | 2.8338 | 0.0036 | 0.0366 | 0.0282 | 5594/5797/387/10627/1495/9411/5787/5792/10398/999/7082/5906/4089/4008/5770/7525/117178/10810/8936/9855/1003/4088/7046/10458/1500/87/998/7048/56288/8826/51176/2260/10580/1457/57154/6591/4233/52/2064/54922/83439/103910/83605/55698/4301/5819/2241/51701 | 48 |
| Human Diseases | Cancer: specific types | hsa05218 | Melanoma | 39/3200 | 73/8541 | 0.534246575 | 1.4259 | 2.8288 | 0.0038 | 0.0379 | 0.0291 | 5594/5156/2252/51426/27006/5296/999/7157/369/1019/5605/4893/8503/1643/1871/578/5293/10912/5155/2246/2254/8817/1029/207/4286/4616/595/3479/2260/3082/4193/4233/5291/80310/10000/1021/673/1870/208 | 39 |
| Genetic Information Processing | Folding, sorting and degradation | hsa03018 | RNA degradation | 41/3200 | 78/8541 | 0.525641026 | 1.4030 | 2.7673 | 0.0045 | 0.0433 | 0.0333 | 29883/9652/84186/54464/170506/51013/1656/54766/10438/3313/23019/7832/9337/23658/6499/10200/51690/11157/10950/5042/23404/9125/23016/22894/80349/26986/56915/55802/51691/4848/10766/87178/80336/246175/131870/4849/219988/132430/2023/3329/167227 | 41 |
| Metabolism | Global and overview maps | hsa01240 | Biosynthesis of cofactors | 73/3200 | 152/8541 | 0.480263158 | 1.2819 | 2.7138 | 0.0047 | 0.0437 | 0.0336 | 221264/23057/122481/4337/7390/64802/51727/2058/4831/2937/217/4830/224/1719/203/790/2729/2356/3145/2235/5373/55312/6697/5372/8836/2730/23475/1371/205/4832/8564/51601/211/8630/7363/7389/5805/9104/204/80308/4833/1723/2677/6472/586/249/51109/29926/210/55191/26289/441024/10243/29968/1355/55163/50808/154807/25902/326625/53354/133686/124454/29922/349565/493911/4351/158067/7360/10797/4338/195814/200895 | 73 |
| Metabolism | Global and overview maps | hsa01232 | Nucleotide metabolism | 44/3200 | 85/8541 | 0.517647059 | 1.3816 | 2.7369 | 0.0048 | 0.0440 | 0.0338 | 221264/144811/122481/1841/4907/957/7083/7298/30833/284958/51727/4831/6240/4830/6241/203/353/9583/132/7084/205/100/4832/5167/956/1854/22978/3704/1716/7371/204/271/4833/8833/51292/79077/26289/54963/5169/51251/50808/29922/131870/158067 | 44 |
| Environmental Information Processing | Signal transduction | hsa04015 | Rap1 signaling pathway | 98/3200 | 212/8541 | 0.462264151 | 1.2338 | 2.6683 | 0.0051 | 0.0449 | 0.0346 | 5594/55740/5156/23683/2252/387/9771/27006/5296/801/2771/999/1942/5899/5590/1398/5906/5605/2776/4893/8503/9693/1436/2263/5293/5332/5909/5155/2261/115/9855/2264/3690/9732/3815/1945/51196/2246/284/11069/2254/7010/2277/4803/2846/8817/207/805/5028/5606/1500/998/3397/5900/3479/5228/5584/9863/27040/5603/56288/7422/2260/3082/5600/2533/6237/83660/5578/5331/4233/1946/2778/3688/5291/10451/64411/80310/2321/10000/260425/4301/5898/9223/2889/673/4254/84552/2324/57121/196883/23566/2903/84612/375189/208/1943/111 | 98 |
| Environmental Information Processing | Signal transduction | hsa04310 | Wnt signaling pathway | 82/3200 | 174/8541 | 0.471264368 | 1.2578 | 2.6596 | 0.0053 | 0.0456 | 0.0350 | 387/6500/894/1488/3725/896/7157/1857/6422/1454/5530/4089/56998/25776/2487/5332/6424/4316/7089/22943/11197/5602/7091/2239/4088/4920/7472/4919/4163/7855/7482/27121/8840/5533/4775/8454/595/23291/4041/1501/27130/2535/51176/1457/818/7090/23500/5578/5331/324/440193/8945/8325/55366/54894/23411/81839/10023/27123/83439/7473/59343/79718/8313/6423/80326/147495/57805/5601/166336/144165/2932/59352/817/85407/80319/64321/85409/51701/284654/4040/1856 | 82 |
| Human Diseases | Cancer: specific types | hsa05224 | Breast cancer | 71/3200 | 148/8541 | 0.47972973 | 1.2804 | 2.6637 | 0.0054 | 0.0456 | 0.0350 | 5594/2252/51426/6654/8202/27006/5296/3725/6464/7157/369/1857/1019/5605/4853/4893/8503/3280/1643/1871/578/6199/5293/10912/672/3815/2246/2099/4855/7472/7855/2254/7482/8817/207/4616/675/595/8648/4041/3479/2535/8600/51176/2260/6655/6667/2885/324/8325/2064/5291/23462/10023/83439/7473/8313/10000/54567/80326/1021/2932/673/26508/1870/5241/53358/2324/208/4040/1856 | 71 |

**Table S3.** Combined Annotated Hub genes

| **ProbeID** | **GeneSymbol** | **Module** |
| --- | --- | --- |
| 213775_x_at | ZNF638 | black |
| 229398_at | RAB18 | black |
| 230337_at | SOS1 | black |
| 232323_s_at | TTC17 | black |
| 242712_x_at | RANBP2 | black |
| 222693_at | FNDC3B | magenta |
| 226265_at | QSER1 | magenta |
| 219640_at | CLDN15 | purple |
| 211178_s_at | PSTPIP1 | red |
| 212415_at | SEPTIN6 | red |
| 219358_s_at | ADAP2 | red |
| 219812_at | PVRIG | red |

**Table S4.** Optimized Hyperparameters

| **Model** | **Optimized Parameters** | **Best CV AUC** |
| --- | --- | --- |
| SVM | sigma = 0.125; C = 2 | 96.9 |
| LDA | parameter = none | 92.39 |
| KNN | k = 13 | 95.42 |
| XGB | nrounds = 100; max_depth = 1; eta = 0.1; gamma = 0; colsample_bytree = 0.8; min_child_weight = 1; subsample = 0.8 | 97.53 |
| LR | alpha = 0; lambda = 0.152641796717523 | 93.17 |

**Table S5. Mean Performance**

| **Model** | **Accuracy_Mean** | **Accuracy_SD** | **AUC_Mean** | **AUC_SD** | **Sensitivity_Mean** | **Sensitivity_SD** | **Specificity_Mean** | **Specificity_SD** |
| --- | --- | --- | --- | --- | --- | --- | --- | --- |
| SVM | 0.929936842 | 0.054473975 | 0.968979798 | 0.039097485 | 0.954182 | 0.060681 | 0.897667 | 0.093976 |
| LDA | 0.855347368 | 0.076200762 | 0.923919192 | 0.058646918 | 0.829091 | 0.129392 | 0.890889 | 0.097281 |
| KNN | 0.880631579 | 0.07638411 | 0.954222222 | 0.05086048 | 0.816727 | 0.128037 | 0.966333 | 0.061793 |
| XGB | 0.900357895 | 0.059448535 | 0.975282828 | 0.027109861 | 0.906909 | 0.088279 | 0.891222 | 0.110673 |
| LR | 0.886631579 | 0.06773243 | 0.931747475 | 0.052199082 | 0.912727 | 0.09193 | 0.851556 | 0.111285 |
